# Countershading in zebrafish results from an Asip1 controlled dorsoventral gradient of pigment cell differentiation

**DOI:** 10.1038/s41598-019-40251-z

**Published:** 2019-03-05

**Authors:** Laura Cal, Paula Suarez-Bregua, Pilar Comesaña, Jennifer Owen, Ingo Braasch, Robert Kelsh, José Miguel Cerdá-Reverter, Josep Rotllant

**Affiliations:** 1 Deparment of Biotechnology and Aquaculture. Instituto de Investigaciones Marinas, IIM-CSIC, Vigo, 36208 Spain; 2 Department of Biology and Biochemistry and Centre for Regenerative Medicine, University of Bath, Claverton Down, Bath, BA2 7AY United Kingdom; 3 Department of Integrative Biology and Program in Ecology, Evolutionary Biology and Behavior, Michigan State University, East Lansing, MI 48824 USA; 4 Instituto de Acuicultura de Torre de la Sal, IATS-CSIC, Castellón, 12595 Spain

## Abstract

Dorso-ventral (DV) countershading is a highly-conserved pigmentary adaptation in vertebrates. In mammals, spatially regulated expression of agouti-signaling protein (ASIP) generates the difference in shading by driving a switch between the production of chemically-distinct melanins in melanocytes in dorsal and ventral regions. In contrast, fish countershading seemed to result from a patterned DV distribution of differently-coloured cell-types (chromatophores). Despite the cellular differences in the basis for counter-shading, previous observations suggested that Agouti signaling likely played a role in this patterning process in fish. To test the hypotheses that Agouti regulated counter-shading in fish, and that this depended upon spatial regulation of the numbers of each chromatophore type, we engineered *asip1* homozygous knockout mutant zebrafish. We show that loss-of-function *asip1* mutants lose DV countershading, and that this results from changed numbers of multiple pigment cell-types in the skin and on scales. Our findings identify *asip1* as key in the establishment of DV countershading in fish, but show that the cellular mechanism for translating a conserved signaling gradient into a conserved pigmentary phenotype has been radically altered in the course of evolution.

## Introduction

Most vertebrates exhibit a dorso-ventral pigment pattern characterized by a light ventrum and darkly colored dorsal regions. This countershading confers UV protection against solar radiation, but also is proposed to provide anti-predator cryptic pigmentation. In mammals, hair color results from biochemical differences in the melanin produced by melanocytes, the only neural-crest derived pigment cell-type in this taxon. Best studied in mice, the local expression of agouti-signaling protein (ASIP) in the ventral skin drives the dorso-ventral pigment polarization^1,2^. ASIP is mainly produced by dermal papillae cells where it controls the switch between production of eumelanin (black/brown pigment) to pheomelanin (yellow/red pigment) by antagonizing α-melanocyte-stimulating hormone (α-MSH) stimulation of the melanocortin 1 receptor (MC1R)^1^. Temporal control of Asip expression as a pulse midway during the hair growth cycle generates a pale band of pheomelanin in an otherwise dark (eumelanin) hair (‘agouti’ pattern). In contrast, in the ventral region, constitutive expression of Asip at high levels represses eumelanin production, resulting in pale hair colour.

Most other groups of vertebrates share the dorso-ventral countershading pattern, but in ray-finned fishes it results from a patterned distribution of light-reflecting (iridophores and leucophores) and light-absorbing (melanophores, xanthophores, erythrophores, and cyanophores) chromatophores^3,4^. Zebrafish, a teleost fish model for pigment studies, obtains its striped pigmentation by the patterned distribution of three types of chromatophores: melanophores, iridophores and xanthophores^5,6^. Furthermore, it is widely accepted that fish melanophores only produce dark eumelanin, but not pheomelanin^7^. Our recent experiments using overexpression systems have demonstrated that zebrafish utilizes two distinct adult pigment-patterning mechanisms, the striped patterning mechanism and the dorso-ventral patterning mechanism^8^. Both patterning mechanisms function largely independently, with the resultant patterns superimposed to give the full pattern^8^. The zebrafish striping mechanism has received much attention and is based on a cell-cell interaction mechanism^9,10^. In contrast, dorso-ventral patterning has been largely neglected, but we have recently provided evidence that it depends on *asip1* expression, and furthermore that this is expressed in a dorso-ventral gradient in the skin directly comparable to that in mammals^8,11,12^. This potential conservation of agouti signaling protein function is fascinating, since it opens up the possibility of a very different cellular mechanism of action in mammals and fish^8,13^. Specifically, we have proposed that Asip1 activity in the ventral skin in zebrafish alters the balance of pigment cell differentiation, through repressing melanophore differentiation^8^.

Studies of Asip1 function in fish to date have relied on gene overexpression approaches, but loss-of-function experiments provide a complementary approach to test the interpretation of those overexpression data. Here, we investigate the *in vivo* functional role of *asip1* in zebrafish by generating *asip1* knockout mutants using clustered regularly interspaced short palindromic repeats (CRISPR)-associated protein-9 nuclease (Cas9) genome engineering tools^14^. We demonstrate that *asip1* knockout mutant zebrafish display a disrupted dorso-ventral pigment pattern characterized, in the ventral region, by an increased number of melanophores and xanthophores accompanied by a severe decrease in the number of iridophores, i.e. a dorsalised pigment pattern. This dorsalisation effect extends also somewhat into the stripes, with the more ventral stripes having melanophore and xanthophore numbers closely resembling their more dorsal counterparts. Our loss-of-function results provide support for our previous hypothesis that *asip1* controls the evolutionarily conserved countershading coloration in fish, but via a distinctive cellular mechanism involving control of differentiation of multiple pigment cell-types.

## Results

### Selection and analysis of induced *asip1* loss-of-function mutations in zebrafish

**Figure 1:**
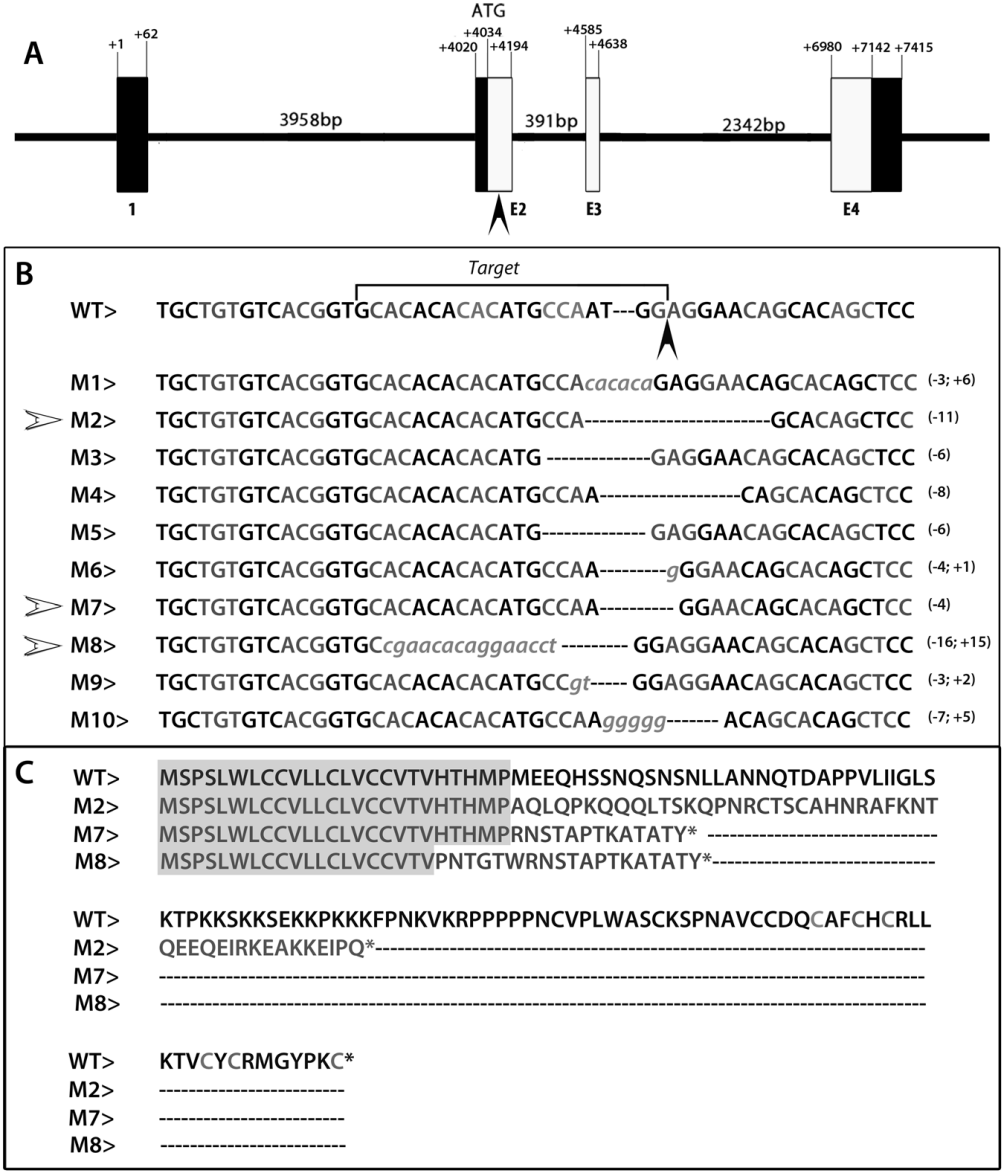
CRISPR/Cas9-induced mutations at the zebrafish *asip1* locus. (**A**) Scheme of the *asip1* gene showing the target site mutation (black arrowhead). Coding exons are represented as white boxes and 5′ UTR and 3′UTR are shown as black boxes. (**B**) Sequence of induced deletions in *asip1* locus. The first line shows the wild-type sequence. Black arrowhead labels the protospacer-adjacent motif (PAM). Next lines show different induced mutations. Italic lower case letters represent inserted new sequence. The number of deleted (−) and inserted (+) bases are marked on the right side of each sequence. Selected mutations are labeled by white arrowheads. (**C**) Predicted amino acid sequence encoded for *asip1* loci. The first line shows the wild type protein, and following lines show the potential protein sequence of each selected mutation. Grey boxes show the wild type sequence. Asterisk represents the stop codon.

Loss-of-function mutations in the *asip1* gene were generated using the CRISPR-Cas9 system. We selected the target site in the first coding exon (60 bp after ATG start codon) (Fig. 1A,B) and found ten different mutated alleles (Fig. 1B). Alleles M1, M3, M5 and M6 conserved the original open reading frame; therefore, they could potentially generate a functional protein lacking only one or two amino acids and keeping almost the entire amino acid sequence. Alleles M2, M4, M7, M8, M9 and M10 show alternative reading frames downstream of the target site. We selected three potential frameshift mutations, which yield predicted nonfunctional proteins. Fish carrying each mutation were raised to generate *asip1*^*K.O*.^ lines (F3 generation) and to characterize the phenotype: M2 (CRISPR1-*asip1*.iim02 zebrafish line), M7 (CRISPR1-*asip1*.iim07 zebrafish line) and M8 (CRISPR1-*asip1*.iim08 zebrafish line) (Fig. 1B). The *asip1*^*iim02*^ allele lacks 11 bp (76–86 bp), the *asip1*^*iim07*^ allele has lost 4 bp (77–81 bp), and *asip1*^*iim08*^ lacks 16 bp (Del 62–76 bp) and carries a 15 bp insertion at position 62 downstream of the predicted ATG start codon (Fig. 1B). In those three alleles, the mutations result in premature stop codons. The *asip1*^*iim02*^, *asip1*^*iim07*^ and *asip1*^*iim08*^ encode 71, 38 and 31 amino acid mutant proteins, respectively (Fig. 1C). All mutated proteins have lost most of their basic central domain and, most significanctly, the C-terminal poly-cysteine domain, which is the crucial region for protein activity^15–17^. All *asip1* knockout mutant zebrafish lines examined resulted in a similar dorso-ventral pigment phenotype as described below.

### *asip1* function in dorsal-ventral pigment patterning

**Figure 2:**
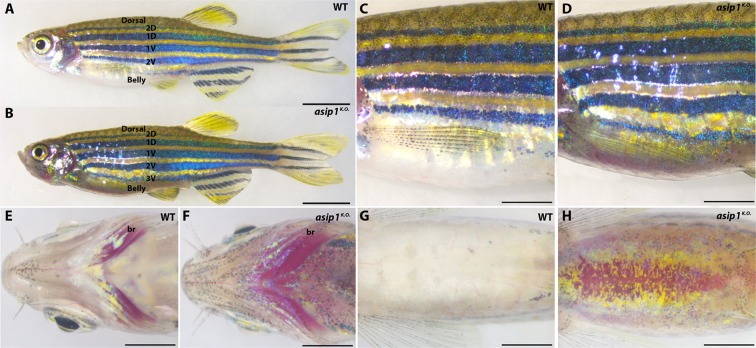
Adult dorso-ventral countershading pattern is disrupted in *asip1*^*K.O*.^. Lateral (**A**,**B**), anterior-lateral (**C**,**D**), ventral head (**E**,**F**) and ventral belly (**G**,**H**) views of 180 dpf WT and *asip1*^*K.O*.^ zebrafish. (**A**,**B**) The pigment pattern of WT zebrafish is a striped pigment pattern with dark stripes and light interstripes. Each dark stripe is named with a code: two primary stripes are called 1D and 1 V, and the two secondary stripes are named 2D and 2 V. The *asip1*^*K.O*.^ display an extra 3 V dark stripe. The *asip1*^*K.O*.^ phenotype is characterized by a darker belly than WT. (**C**,**D**) The striped pigment pattern was almost unaltered in *asip1*^*K.O*.^ fish, except that the 2 V stripe is wider than in WT, and the ventral dark stripe 3 V is better developed anteriorly. The darker belly of *asip1*^*K.O*.^ compared to WT sibling fish is clearly evident. (**E**,**F**) In WT, melanophores are infrequent around the jaws and branchiostegals; however, branchiostegal, jaw and operculum regions are clearly hyperpigmented in *asip1*^*K.O*.^. (**G**,**H**) Melanophores are virtually absent in WT belly; thus, WT ventral region shows bright white color as a result of high number of iridophores in the abdominal wall. However, *asip1*^*K.O*.^ shows a consistent hyperpigmentation, with many melanophores and xanthophores in the ventral skin; the abdominal wall also seems to be affected, with reduced extent of iridophores and looking much yellower than WT. Scale bar: (**A**,**B**) 5 mm, (**C**–**H**) 2 mm. Abbreviation: br, branchiostegal.

**Figure 3:**
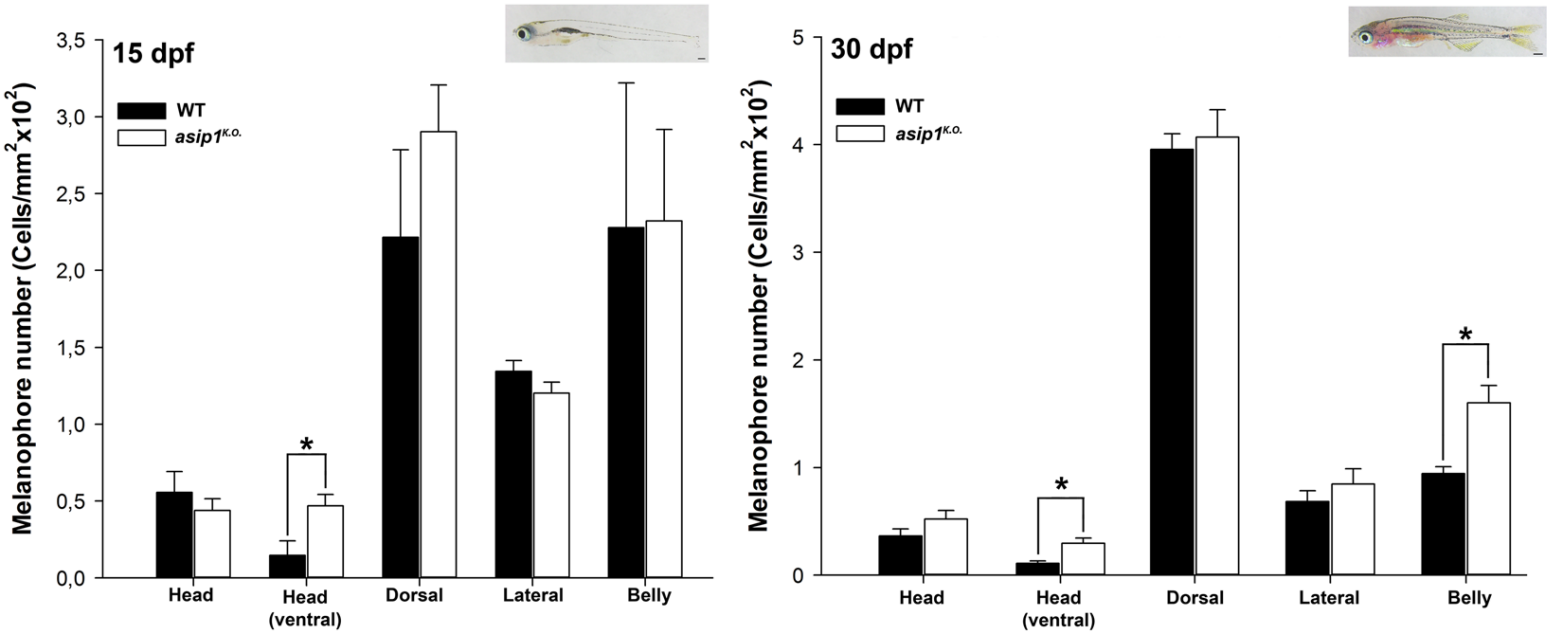
Dorsal-ventral distribution of melanophores during metamorphosis. (**A**) Distribution and number of melanophores in 15 dpf WT and *asip1*^*K.O*.^ fish. At this stage, *asip1*^*K.O*.^ already shows significantly higher number of melanophores in the ventral view of the head. (**B**) Distribution and number of melanophores in WT and *asip1*^*K.O*.^ 30 dpf fish. At this stage, *asip1*^*K.O*.^ shows significantly higher number of melanophores in the ventral view of the head, but also in the belly. Data are the mean ± SEM, n = 7. Asterisks indicate significant differences between WT and *asip1*^*K.O*.^ fish. Scale bar: (**A**) 200 μm, (**B**) 500 μm.

**Figure 4:**
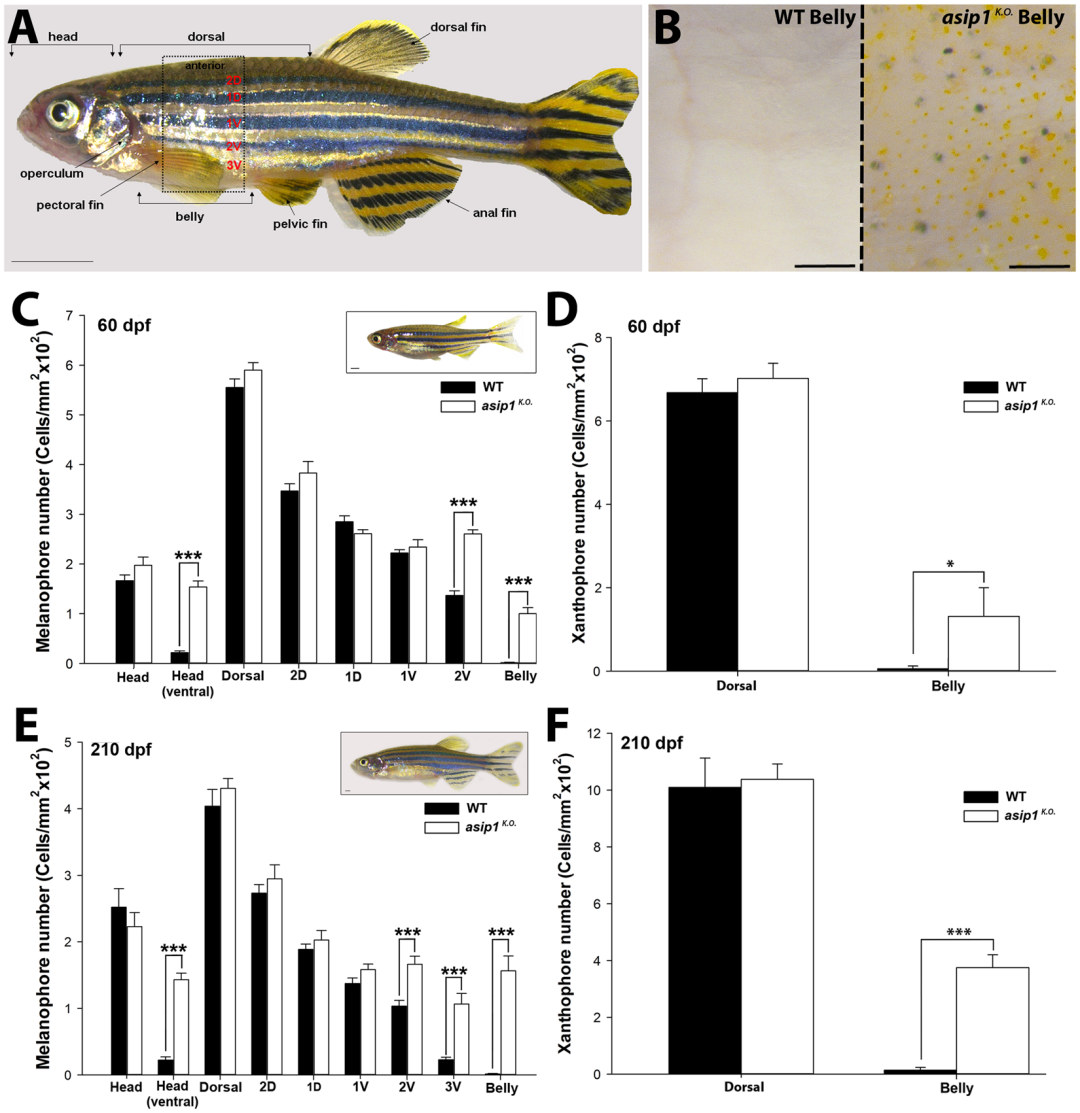
Quantitation of dorsal-ventral distribution of melanophores and xanthophores in adult WT and *asip1*^*K.O*.^ fish. (**A**) Lateral view of zebrafish showing the body regions selected for melanophore and xanthophore count. (**B**) Ventral view of the WT and *asip1*^*K.O*.^ 210 dpf zebrafish fish belly. (**C**) Distribution and number of melanophores in WT and *asip1*^*K.O*.^ 60 dpf fish. At this stage, *asip1*^*K.O*.^ shows a significantly higher number of melanophores in the black stripe 2 V, ventral head and belly. (**D**) Number of xanthophores in the dorsal and ventral skin of WT and *asip1*^*K.O*.^ 60 dpf fish. At this stage, *asip1*^*K.O*.^ shows a significantly higher number of xanthophores in the belly region. (**E**) Distribution and number of melanophores in WT and *asip1*^*K.O*.^ 210 dpf fish. At this stage, *asip1*^*K.O*.^ shows significantly higher number of melanophores also in black stripe 2 V, 3 V, ventral head and belly. (**F**) Number of xanthophores in dorsal and ventral skin of WT and *asip1*^*K.O*.^ 210 dpf fish. These fish showed highly significant higher number of xanthophores in belly region than WT. Data are the mean ± SEM, n = 7. Asterisks indicate significant differences between WT and *asip1*^*K.O*.^ fish. Scale bar (**A**,**C**,**E**) 1 mm, (**B**) 100 μm.

All three *asip1*-CRISPR knockout lines exhibited a loss of dorso-ventral countershading. Because we did not find any difference in the pigment pattern across the three-knockout mutants’ lines, we focused on the study of line CRISPR1-*asip1*.iim08, here referred to as *asip1*^*K.O*.^. In *asip1*^*K.O*.^ fish, melanophores and xanthophores were more numerous in all ventral regions (Fig. 2A–D), including the ventral head (Fig. 2E,F). In WT fish, melanophores and xanthophores were very limited in the ventral region, and mainly located on the jaw and the posterior belly regions, near the pelvic fins (Fig. 2G). The WT phenotype shows a low number of melanophores in the ventral head region and high number of iridophores around the branchiostegals and operculum (Fig. 2E). In contrast, *asip1*^*K.O*.^ mutants show melanophores spread throughout the jaws, branchiostegal and opercular regions (Fig. 2F). On the belly, the ventral skin of WT fish showed almost a total absence of melanophores, so that the bright whitish-reflective iridophore sheet of the internal abdominal wall is prominent (Fig. 2G). Conversely, *asip1*^*K.O*.^ fish displayed a strong increase in melanophore and xanthophore number in the ventral skin, as well as many extra cells that transform the incipient 3 V of the WT into a prominent 3 V reaching to the head in the *asip1*^*K.O*.^ (Fig. 2A–D). We note that the consistent increase in melanophore numbers in the 2 V and 3 V stripes can also be considered a dorsalisation phenomenon, since our counts show them to now resemble their more dorsal counterparts (Figs 3 and 4). In addition, the abdominal wall exhibits an obvious decrease in the number of iridophores, resulting in an apparent breakup of the iridophore sheet into smaller fragments, thus conferring a darker color to the ventral region of *asip1*^*K.O*.^ fish (Fig. 2H). The Sanger-generated mutant, *asip1*^*sa13993*^, showed only a subtle and partial phenotype compared to *asip1*^*K.O*.^ fish, ((e.g. hyperpigmentation in the belly was not obvious; Supp. Fig. 2)), however, the incipient 3V-stripe of the WT becomes more fully developed in the *asip1*^*sa13993*^ mutant line.

### Development of the zebrafish *asip1*^*K.O*.^ phenotype

To establish the time point when the phenotype of the *asip1* mutants (*asip1*^*K.O*.^) becomes first apparent during development, melanophores were counted at larval (5 dpf, SL 3 mm), metamorphic (15 dpf, SL 6.3 mm and 30 dpf, SL 7 mm) and two adult stages (60 dpf, SL 13 mm and 210 dpf, SL 25 mm) (Figs 3 and 4). It has been shown that pigment pattern changes during development can be distinguished by an increase in the melanophore number and changes in their distribution^18,19^. We have quantified the distribution of melanophores in WT and *asip1*^*K.O*.^ fish along the dorsal-ventral axis, by sampling at defined positions in the dorsal and ventral head, lateral stripe, and belly (see Materials and Methods and Figs 3 and 4 for details). No differences in melanophore numbers were found at larval stages (5 dpf, SL 3 mm) (data not shown). In contrast, the dorsal-ventral pigment abnormalities began to be visible from the earliest stages of metamorphosis (15 dpf, SL 6.3 mm). Although at 15 dpf there were no differences in melanophore number in the belly between *asip1*^*K.O*.^ and WT fish, melanophore number in the ventral head was 68.7% higher in *asip1*^*K.O*.^ fish than in WT fish (P < 0.05) (Fig. 3A). At 30 dpf, pigment abnormalities also appear in the belly: melanophore number in the ventral head was 63% higher in the *asip1*^*K.O*.^ than in WT fish (P < 0.05), while in the belly melanophore numbers were 41% higher in *asip1*^*K.O*.^ than WT belly (P < 0.05) (Fig. 3B).

The *asip1*^*K.O*.^ fish at 60 and 210 dpf showed significant pigment pattern alterations, particularly in the ventral region compared to WT fish (Fig. 4B). At 60 dpf, the number of skin melanophores of *asip1*^*K.O*.^ fish was 47% higher (P < 0.001) in dark stripe 2 V, 86% higher (P < 0.001) in the ventral head, and 98% higher (P < 0.001) in the belly than in equivalent positions of WT fish. No differences were found in dorsal regions or in other dark stripes (Fig. 4C). Furthermore, we found that the number of xanthophores was also affected in ventral regions. At 60 dpf, the distribution of xanthophores in anterior area of the belly was 98% higher (P < 0.05) than in WT. No differences were found in dorsal regions (Fig. 4D). At 210 dpf, the same pattern of an increased number of melanophores in the ventral region was found. The number of melanophores in *asip1*^*K.O*.^ fish was 38% higher (P < 0.001) in dark stripe 2 V, 78.6% higher (P < 0.001) in dark stripe 3 V, 84% higher (P < 0.001) in the ventral head, and 99% higher (P < 0.001) in the belly compared to the equivalent region of WT siblings. Just as in 60 dpf fish, the pigment defects were restricted to ventral regions (Fig. 4E). At 210 dpf, the number of xanthophores in the belly region was 96% higher (P < 0.001) compared to WT siblings, while no differences were found in dorsal regions (Fig. 4F).

**Figure 5:**
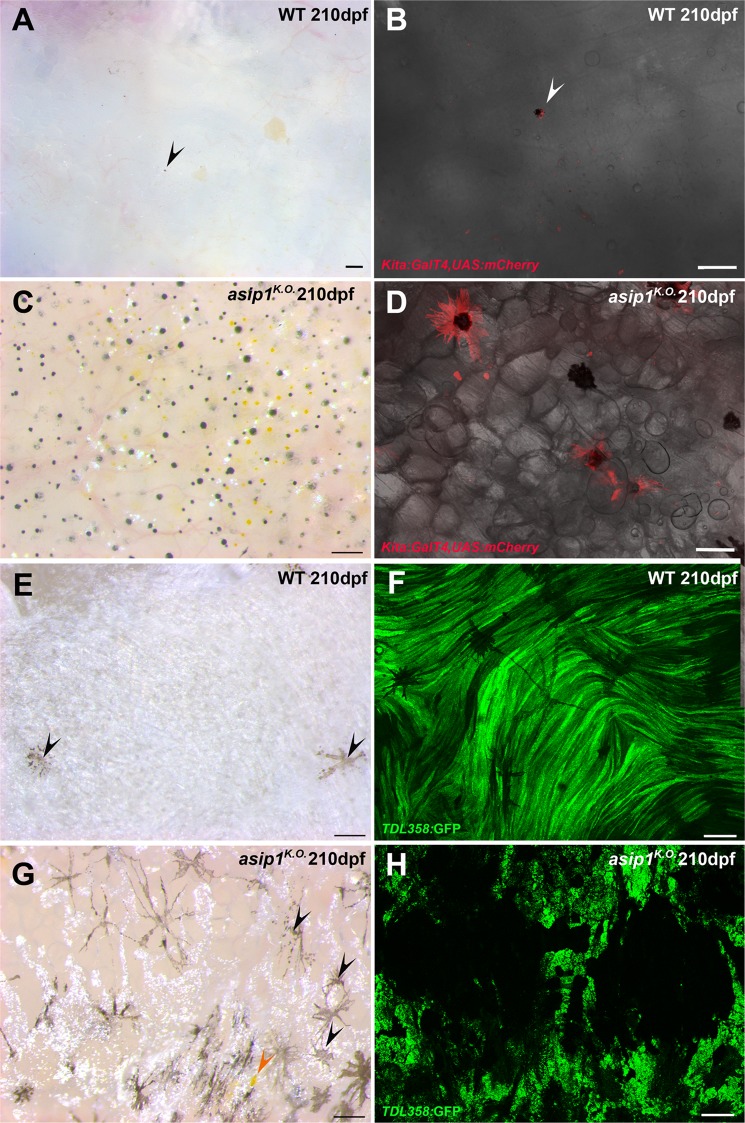
Detailed visualization of ventral pigment cells in WT and *asip1* mutants. (**A**) Ventral view of 210 dpf WT belly. (**B**) Belly of 210 dpf WT fish carrying *Tg(Kita:GalTA4;UAS:mCherry*) (labels melanophores) transgene shows no melanophores in ventral skin. (**C**) Ventral view of 210 dpf *asip1*^*K.O*.^ belly. (**D**) Belly of 210 dpf *asip1*^*K.O*.^ fish carrying *Tg(Kita:GalTA4;UAS:mCherry*) transgene shows high number of melanophores in ventral skin. (**E**) Internal view of 210 dpf WT abdominal wall shows a white sheet of iridophores with few internal melanophores (black arrow). (**F**) Abdominal wall of 210 dpf WT fish carrying *Tg(TDL358:GFP)* (labels iridophores and glia) transgene displays a uniform and continuous sheet of iridophores. (**G**) Internal view of 210 dpf *asip1*^*K.O*.^ abdominal wall shows a disrupted and discontinuous sheet of iridophores with high number of melanophores (black arrow) and some xanthophores (orange arrow). (**H**) Abdominal wall of 210 dpf *asip1*^*K.O*.^ fish carrying *Tg(TDL358:GFP)* transgene exhibits a broken sheet of iridophores. Scale bars: 100 μm.

If Asip1 functioned in fish by a homologous cellular mechanism to that in mammals, we would predict the presence of unpigmented melanophores in the ventral skin. To test this, and to supplement the analysis of pigment cells using their autonomous pigmentation, we also compared the distribution of transgenic markers of melanophores and iridophores in *asip1*^*K.O*.^ mutants and their WT siblings. Firstly, we imaged fish carrying the *Tg(Kita:GalTA4,UAS:mCherry)* transgene, which labels melanophores with membrane-bound mCherry^20^. In WT, melanophores were almost never detected in ventral skin region (Fig. 5A), but importantly neither were unpigmented mCherry-expressing cells (Fig. 5B). In contrast, *asip1* mutants displayed many transgenically-labelled melanophores in the ventral skin region (Fig. 5C,D). This is in agreement with the observed increase in the number of melanophores in *asip1*^*K.O*.^ at later stages of development (Fig. 4), but extends those observations to argue against the presence of specified but amelanic melanophores in the WT belly.

By analyzing fish carrying *Tg(TDL358:GFP)* transgene, which label iridophores and glia with cytosolic GFP^21^, we confirmed the dense and uniform sheet of iridophores in the abdominal wall of WT fish (Fig. 5E,F) and showed that, this sheet is broken up into small fragments in *asip1*^*K.O*.^ mutants (Fig. 5G,H). Thus, *asip1*^*K.O*.^ mutants showed a strong reduction of the iridophore number and many interspersed melanophores (Fig. 5G, black arrow), as well as some xanthophores (Fig. 5G, orange arrow) in the abdominal wall.

**Figure 6:**
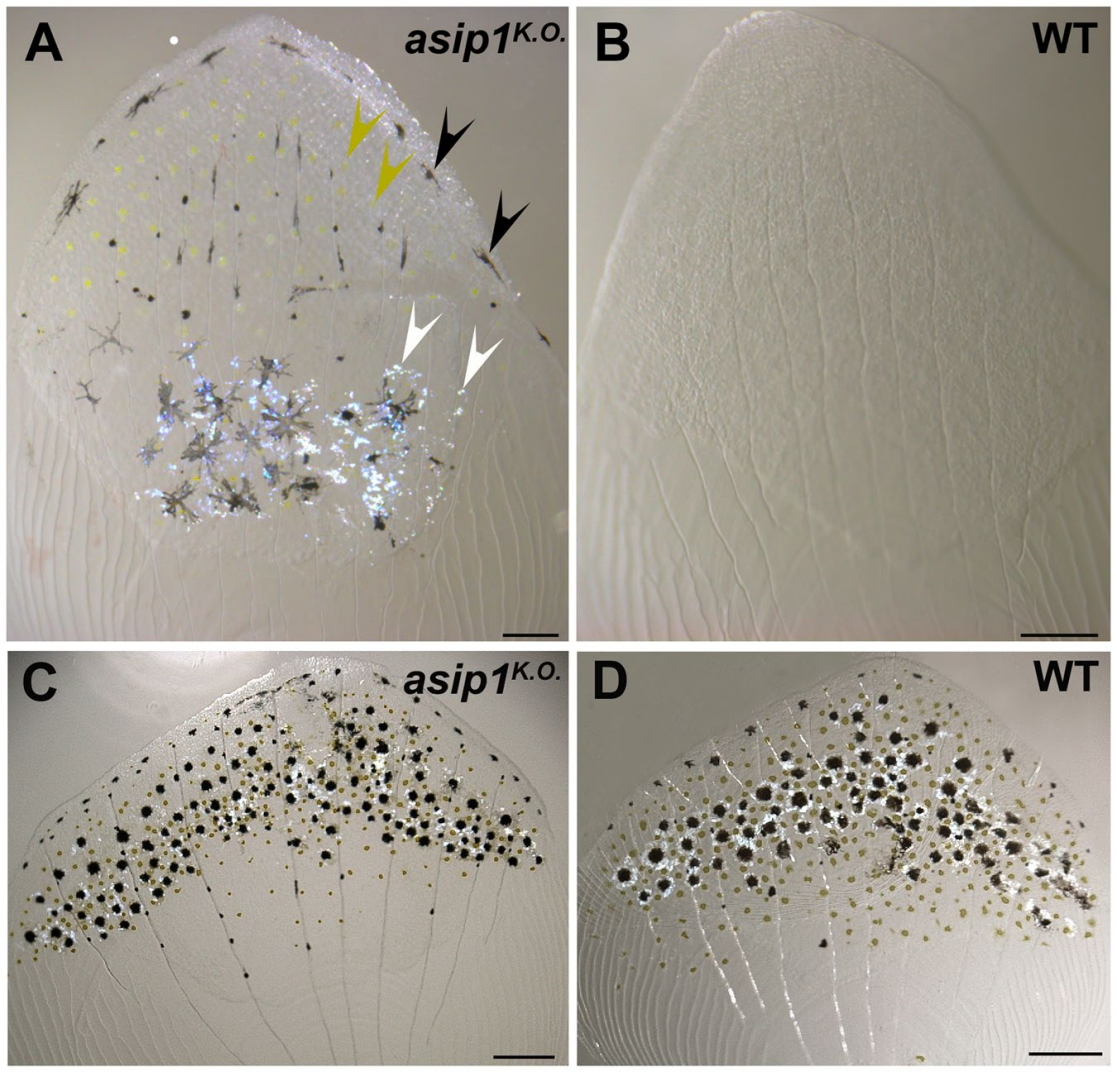
Adult *asip1*^*K.O*.^ ventral scales displayed a dorsalized color pattern. (**A**) 210 dpf *asip1*^*K.O*.^ ventral scales exhibit a pattern of melanophores (black arrowheads), xanthophores (yellow arrowheads) and also iridophores (white arrowheads). (**B**) 210 dpf WT ventral scale does not exhibit any chromatophores. (**C**,**D**) 210 dpf WT and *asip1*^*K.O*.^ dorsal scales exhibit a similar pattern of melanophores, xanthophores and iridophores. Scale bars: 100 μm.

Additionally, we characterized the contribution to the disrupted countershading phenotype in *asip1*^*K.O*.^ mutants of pigment cells in the scales. In contrast to ventral scales of WT siblings which lack all pigmented cell-types (Fig. 6B), ventral scales of *asip1* mutants displayed numerous melanophores (Fig. 6A, black arrowheads), xanthophores (Fig. 6A, yellow arrowheads) and extensive silvery patches of iridophores (Fig. 6A, white arrows). Thus, scales isolated from the belly of *asip1* mutants displayed a “dorsalized” color pattern (*i.e*., ventral scales become nearly as dark colored as dorsal scales due to an increased number of pigment cells) (Fig. 6C,D).

### Rescue of CRISPR mediated mutations

**Figure 7:**
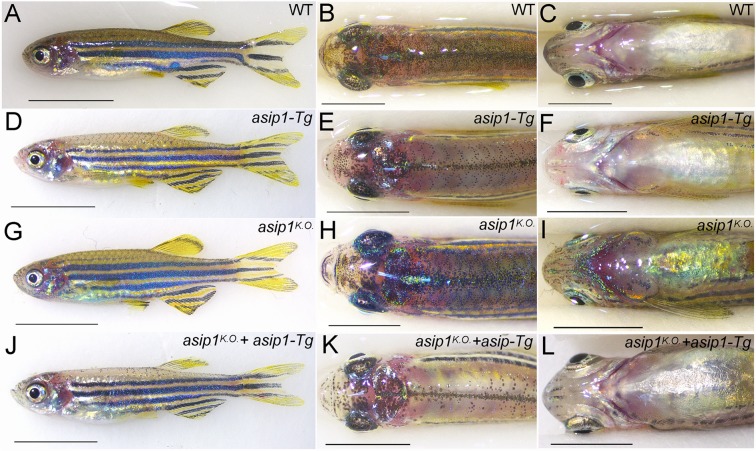
Functional rescue of CRISPR-mediated *asip1* mutation. Lateral (**A**,**D**,**G**,**J**), dorsal (**B**,**E**,**H**,**K**) and ventral-belly (**C**,**F**,**I**,**L**) views of 160 dpf WT, *asip1*-Tg, *asip1*^*K.O*.^, and *asip1*^*K.O*^; *asip1*-Tg zebrafish. The pigment pattern of WT zebrafish shows (**A**) normal striped pattern, (**B**) dark dorsum and (**C**) light belly. The pigment pattern of *asip1*-Tg fish shows (**D**) almost normal striped pattern, although dark stripe 2D??? is rather thinner???, (**E**) hypopigmented dorsum and (**F**) light belly. The pigment pattern of *asip1*^*K.O*.^ fish shows (**F**) almost normal striped pattern, but with dark stripes 2 V and 3 V more developed than WT fish, (**H**) pigmented dorsum similar to WT and (**I**) hyperpigmented belly compared to WT. The *asip1*^*K.O*^ + *asip1* − Tg phenotype shows a phenotype similar to the *asip1* − Tg zebrafish, except that dark stripe 2D is more prominent. Scale bar: 5 mm.

Finally, as a key test of our model, we assess the effect of combining the knockout (KO) mutant with our previously-described *asip1*-Tg zebrafish line overexpressing *asip1* in the entire body. In our model, a graded distribution of Asip1 controls the ratio of melanophore, xanthophore and iridophore differentiation in the skin, with high levels ventrally characteristically repressing melanocyte and stimulating iridophore differentiation; in the dorsum, where Asip1 levels are lowest, melanophores differentiate and iridophores are suppressed. We have shown that our *asip1-Tg* line shows a strongly ventralised pigment pattern in the dorsum (Fig. 7D–F; reference), suggesting that the ubiquitous Asip1 levels generated are equivalent to those in the belly region of a WT fish. We predict therefore that in the background of our new *asip1*^*KO*^ which lacks the endogenous gradient of Asip1, the pigment pattern should also be ventralised, but might, if anything, show a slightly weaker phenotype due to the absence of endogenous Asip1 ‘supplementing’ the transgenic Asip1 expression. This is indeed what we observed (Fig. 7). WT fish show the typical striped pattern (Fig. 7A), combined with a darker dorsum (Fig. 7B), and a light ventrum (Fig. 7C). The *asip1*-Tg zebrafish phenotype presents a striped pattern that shows a mild reduction in melanophore number in the 1D and 2D stripes (Fig. 7D), a light belly similar to WT fish (Fig. 7F), but a drastic reduction of dorsal melanophores (Fig. 7E) due to the ectopic overexpression of *asip1*^8^. In *asip1*^*K.O*.^ mutants (Fig. 7G) the striped pattern is enhanced, with a prominent 3 V stripe reaching to the head (Fig. 7F), the belly is considerably darker (dorsalised) than in WT (Fig. 7I), while the dorsum remains similar to that of WT (Fig. 7H). In the asip*1*^*K.O*.^; *asip1-*Tg, the *asip1*^*K.O*.^ phenotype is suppressed and the *asip1*-Tg^.^ phenotype prevails (Fig. 7J). The *asip1*^*K.O*.^*; asip1*-Tg zebrafish do not show enhancement of the 3 V stripe, but instead show a stripe pattern similar to the *asip1*-Tg^.^, except that the ?2D stripe is somewhat more prominent, due to a more WT melanophore count (Fig. 7J), a light dorsum with a drastic reduction of dorsal melanophore as the *asip1*-Tg^.^ fish (Fig. 7K), but a light belly similar to both *asip1*-Tg and WT fish (Fig. 7L). These observations are fully consistent with our hypothesis that the graded expression of *asip1* along the dorso-ventral axis is crucial to establish the dorso-ventral pigment pattern and that this results from changed numbers of multiple pigment cell-types.

## Discussion

*Asip* is a key gene regulating mammalian countershading. Ubiquitous expression of *Asip* in viable agouti yellow mice (*A*^*y*^) results in a phenotype characterized by yellow fur, as well as hyperphagia, obesity and increased linear growth^22,23^. Mammalian countershading results from an asymmetry in the dorsoventral axis of *Asip* expression in the skin, with high levels in ventral regions being driven by a constitutively active promoter^1^. Similarly, transgenic *asip1* overexpression in zebrafish also results in a disruption of the dorso-ventral pigment pattern^8^, again associated with hyperphagia and increased linear growth^24^. However, the cellular mechanisms leading to the pigment pattern phenotype have been proposed to be different in mammals and fish^8^. In mice, Agouti expression blocks MC1R activity in the ventral skin resulting in a switch in the melanin sub-type being expressed. Thus, constitutive production of ASIP (e.g. in *A*^*y*^ genotypes) drives pheomelanin synthesis at the expense of eumelanin and so results in all yellow fur^22,23^. Conversely, absence of ASIP at all stages of the hair cycle mimics the constitutively active MC1R phenotype, resulting in full eumelanisation of the hair (in place of any agouti-style banding pattern). In zebrafish, ubiquitous overexpression of *asip1* inhibits dorsal melanogenesis and melanophore differentiation but has no major effects on stripe melanophores^8,11,12^. These effects are probably mediated through Mc1r, since this receptor binds Asip1 and agouti-related protein (Agrp) as both competitive antagonists and inverse agonists^11,25^. Alterations in the Mc1r coding sequence cause reduced pigmentation or *brown* phenotypes (reduced number of melanophores and melanin content) in cavefish (*Astianax mexicanus*) whereas Mc1r-morpholino knockdown in zebrafish recapitulates the *brown* pigmentation phenotype^26^. In our previous gain-of-function study, we provided data showing that melanophore differentiation was reduced in the ventralized dorsal regions of *asip1* overexpressing transgenic fish, suggesting that Asip1 represses melanophore differentiation, and *mitfa* expression data consistent with a reduction in melanophore specification too^8^. Our *asip1* loss-of-function data here provides compelling support for this hypothesis that pigment cell fate choice is, in part, regulated by Asip1. *Asip1* knockout lines exhibit a profound increase in number of ventral melanophores, particularly in the ventral region of the head but also along the ventral trunk. This dorsalisation phenomenon extends also to the ventral-most stripes, with the incipient 3V-stripe of the WT becoming fully developed and the 2V-stripe thickened in *asip1*^*K.O*.^ mutant lines. Furthermore, our use of transgenic reporters for melanoblasts and iridoblasts strongly supports the interpretation that these changes result from switching in the types of pigment cells produced in the belly; thus, the phenomenon involves regulation of fate specification from multipotent progenitors, rather than from enhanced or repressed differentiation of specified progenitors.

Using quantitation of expression of the xanthophore and iridophore markers, xanthine dehydrogenase (*xdh*) and leucocyte tyrosinase kinase (*ltk*) respectively^27,28^, we were unable to demonstrate clearly an effect on xanthophore and iridophore differentiation in transgenic *asip1* overexpressing fish^8^. However, these Asip1 transgenic zebrafish did show an extra iridophore interstripe over D1 that we initially interpreted as simply due to the enhanced visibility of underlying iridophores resulting from the lack of melanized cells in the dorsal region^8^. Our new loss-of-function mutants and the rescue of CRISPR induced Asip1 mutations data clearly demonstrates that Asip1 also plays a key role in regulating both iridophore and xanthophore differentiation in the adult skin, suggesting that the extra dorsal iridophore interstripe in Asip1 transgenic fish may, in fact, result from ectopic production of iridophores as well as the absence of melanophores.

Our new loss-of-function data provide independent support for our suggestion^8^ that Asip1 has no role in embryonic pigment cell development nor in larval (pre-metamorphic) pigment pattern formation. However, Asip1-dependent effects on pigment pattern become visible from the very earliest stages of metamorphosis (15 dpf), and then progressively spread to all ventral pattern elements as they are formed during metamorphic growth. We note that the timing of initiation of these effects corresponds to the period when *asip1* expression reaches maximum levels (at 15 dpf) and when significant dorso-ventral differences in *asip1* expression appear (30 dpf^8^). Thus, *asip1* has a role exclusively in metamorphic and post-metamorphic pigment pattern formation.

Early experimental data in amphibian and fish species identified a diffusible melanization inhibition factor (MIF), mainly produced by cells in the ventral skin, that inhibits melanoblast differentiation, but also stimulates or supports iridophore proliferation in the ventrum^29–31^. Our demonstration that absence of Asip1 results in a severe impairment of ventral iridophore development strongly supports the identification of Asip1 as the elusive MIF.

Zebrafish iridophores contribute to silver- or white-colored regions. They are classified into two different types according to the size and number of guanine platelets. Type S iridophores contain smaller uniform-sized platelets, but in larger numbers, than type L iridophores. The abdominal wall is covered by a dense internal sheet of type S iridophore^5,6^. By analyzing *Tg(TDL358:GFP)/asip1*^*K.O*.^ mutant zebrafish lines, we show that Asip1 loss-of-function strongly disrupts this abdominal wall iridophore sheet in the ventral trunk. Our previous studies showed *asip1* expression in the iridophores of the zebrafish abdominal wall by *in situ* hybridization^8^ and promoter-directed reporter expression^13^; our new data suggests that *asip1* is necessary for the normal development of this abdominal iridophore sheet.

**Figure 8:**
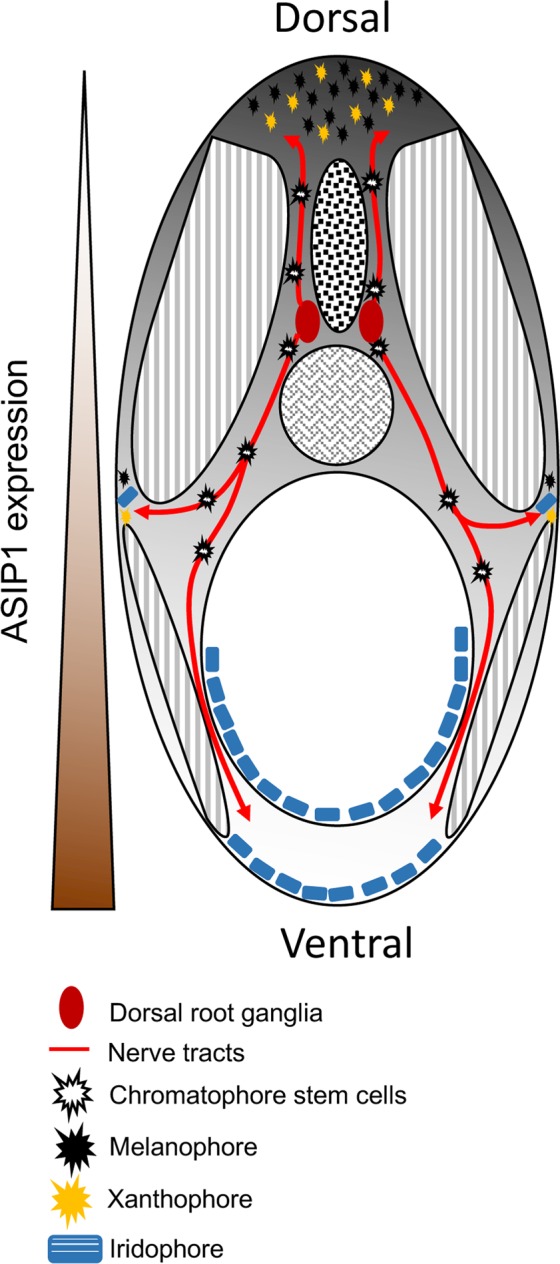
Schematic section of metamorphic zebrafish showing the effect of graded ASIP1 levels on chromatophore specification from multipotent progenitors. Progenitors are delivered to the skin from multipotent stem cells in the DRG via segmental nerves (Singh *et al*.^32^).

It will be important to determine where, and on what cell-type, Asip1 acts to regulate numbers of each pigment cell-type. Melanocyte stem cells identified in the dorsal root ganglia (DRG) have been shown to generate all three pigment cell-types in the post-metamorphic skin of zebrafish, supporting the idea of a common pigment progenitor^32^. These multipotent progenitors have been proposed under a progressive fate restriction model to subsequently segregate bipotent progenitors (melanophore-iridophore, melanophore-xanthophores and xanthophore-iridophore) from which individual pigment cell fates become specified^32^. We propose that Asip1 levels in the skin may control the fate specification of these progenitors when they arrive at the skin. Thus, high ventral levels of Asip1 repress melanophore and xanthophore specification and promote iridophore specification from these progenitors. In contrast, those progenitors choosing the dorsal migratory route from DRG enter a low Asip1 environment and more frequently become melanophores and xanthophores (Fig. 8).

We have shown a dramatic increase in the number of ventral xanthophores in *asip1*^*K.O*.^ mutants. Our original studies identifying Asip1 in fish suggested an effect on xanthophore physiology^11^. Thus, xanthic goldfish, lacking melanophores, also exhibit a dorso-ventral pigment pattern with no xanthophores in the ventral region where *asip1* expression levels are maximal^11^. Our knockout mutant and the rescue of the CRISPR mediated Asip1 mutations studies reinforces the hypothesis that high Asip1 in ventral skin represses xanthophore development.

Dorsalisation of pigment pattern is most striking in the ventral scales in *asip1*^*K.O*.^ compared with WT siblings. Scales on the belly of WT fish lack all chromatophores but surprisingly belly scales in *asip1*^*K.O*.^ exhibit all three types of chromatophores. Although, it has been shown that the effect of Asip1 over iridophores seems to be different in scales and in the skin^29–31^, our data together demonstrate that Asip1 is strongly inhibitory to chromatophore differentiation in the scales. Accordingly, it has been demonstrated that goldfish Asip1 conditioned medium represses medaka scale pigmentation^11^. Scale pigmentation has been less-well studied in zebrafish, but it is thought that multipotent pigment cell progenitors that populate the skin also populate the scales^32^. Further work will be necessary to understand the different responses to Asip1 of these progenitors in scales versus the skin, but we suggest that these reflect an evolutionarily ancestral dorsal countershading mechanism that functions in association with scales, and an evolutionarily derived secondary striping mechanism in deeper layers of the skin.

In conclusion, our loss-of-function experiments support and extend the results from our overexpression analysis showing that the graded expression of *asip1* along the dorso-ventral axis is crucial to establish the dorso-ventral pigment pattern in ray-finned fish. Asip1 has a dramatic effect on the ancestral dorso-ventral pigment patterning process, but also a more subtle control of the striping mechanism. We propose that the Asip1 gradient is an environmental cue that uses the melanocortin-signaling system to bias the adoption of pigment cell fates from progenitors that migrate into the skin (Fig. 8). Interestingly, these biases are subtly different in the scales (where Asip1 represses all pigment cell specification) and the striped skin (where melanophores and xanthophores are repressed, while iridophores are promoted). Our work thus provides an important contribution to understanding how Asip-induced differential effects of cell environment controls pigment cell fate choice from progenitors.

## Methods

### Fish

Zebrafish were reared as previously described^33^ and staged according to Kimmel *et al*.^34^. Fish of the following genotypes were used: TU strain (Tübingen, Nüsslein-Volhard Lab), *Tg(TDL358:GFP)*^21^ and *Tg(kita:GalTA4:UAS:mCherry)*^20^. Fish care and procedures in the Kelsh lab were approved by the University of Bath Ethical Review Committee, and were performed in compliance with the Animals Scientific Procedures Act 1986 of the UK. In the Rotllant lab, ethical approval (Ref.: CSIC/OH-150/2014) for all studies was obtained from the Institutional Animal Care and Use Committee of the IIM-CSIC Institute in accordance with the National Advisory Committee for Laboratory Animal Research Guidelines licensed by the Spanish Authority (RD53/2013). All studies conformed to European animal directive (2010/63/UE) for the protection of experimental animals.

### Generation and analysis of *asip1* knockout mutants

Initial study of *asip1 (sa13992)*, a randomly induced point mutation predicted to affect splicing, failed to reveal a clear pigment pattern defect (Supp. Figs 1 and 2). The *asip1*^*sa13992*^ allele was generated by random mutagenesis during a large-scale mutagenesis project at the Sanger Institute^35^, and obtained from the European Zebrafish Resource Center.

Due to uncertainties about the likely effect of compensatory mechanisms limiting the impact of the predicted change in splicing in *asip1*^*sa13992*^, we to used CRISPR/Cas9 genome editing to engineer a likely null allele. To this end, an *asip1* loss-of-function mutation was generated using a CRISPR-Cas9 protocol originally adapted from Bassett *et al*.^14^ and kindly provided by Dr. Sam Peterson (University of Oregon). The potential target sequence was identified with the ChopChop web tool^36^. Two long oligonucleotides (Scaffold oligo: 5′-GATCCGCACCGACTCGGTGCCACTTTTTCAAGTTGATAACGGACTAGCCTTATTTTAACTTGCTATTTCTAGCTCTAAAAC-3′, and gene-specific oligo 5′- AATTAATACGACTCACTATAGCACACACACATGCCAATGGGTTTTAGAGCTAGAAATAGC-3′) were used to perform a DNA-free PCR to obtain a 125 bp DNA fragment that includes the previously identified target site sequence (5′- GCACACACACATGCCAATGG-3′). The PCR reaction was performed in 20 μL containing 10 μL of 2x Phusion High-Fidelity PCR Master Mix Buffer (New England Biolabs, UK), 1 μL of gene specific oligo (10 μM), 1 μL of gRNA scaffold oligo (10 μM) and H_2_O nuclease free to 20 μL. PCR conditions were 98 °C for 30 sec, 40 cycles of 98 °C for 10 sec, 60 °C for 10 sec, 72 °C for 15 sec, and a final step of 72 °C for 10 min. The PCR product was purified using DNA Clean&Concentration-5 Kit (Zymo Research, USA) according to the manufacturer’s instructions. Purified PCR product was used as template for *in vitro* transcription with MEGAscript T7 High yield transcription Kit (Ambion, USA) according to the manufacturer’s instructions. The gRNA was purified with RNA Clean&Concentrator 5 (Zymo Research, USA) before to use it. Subsequently, the gRNA was injected in a concentration of 25 ng/µL together with Cas9 mRNA (transcribed from the linearized pT3TS-nCas9n plasmid) in a concentration of 50 ng/µL and Phenol red solution (0,1%). Around 2 nL of this mix was microinjected into the cytoplasm of zebrafish eggs at the one- or two-cell stage. Dissection microscope (MZ8, Leica) equipped with a MPPI-2 pressure injector (ASI systems) was used for microinjection. Different mutations were found and three different potential nonfunctional mutations were raised as different *asip1* knockout lines. The phenotype in each knockout stable line was similar. For microscope imaging, zebrafish of 5 dpf, 15 dpf, 30 dpf and 180 dpf were anesthetized with tricaine methasulfonate (MS-222, Sigma-Aldrich) and scales were isolated from the belly and immersed in PBS on a glass slide. Scales and fish were photographed with a Leica M165FC stereomicroscope equipped with a Leica DFC310FX camera.

Double reporter transgenic/*asip1* mutant lines were obtained by setting up crosses between the *asip1* mutant line and a reporter transgenic line *Tg(TDL358:GFP)*, which labels iridophores^21^, or a reporter transgenic line *Tg(kita:GalTA4:UAS:mCherry)*, which labels melanophores^20^. The offspring of these crosses were incrossed to obtain homozygous *asip1* knockout mutants. Imaging was carried out on a Leica TCS SP5 confocal microscope. 5 dpf, 15 dpf and 30 dpf transgenic zebrafish were anesthetized and photographed. Adult zebrafish (180 dpf) were anesthetized with MS-222 and decapitated to sample a ventral skin section including the abdominal wall and ventral and dorsal scales. Skin section and scales were placed in PBS and photographed.

### Melanophore and xanthophore counts

The melanophore pattern of *asip1* knockout mutant fish (*asip1*^*K.O*.^) was compared with that of the control fish by quantification of melanized melanophores in both groups (Fig. 2). The selected regions for melanophore counts were different at each stage of development. At the early larval stage (5 dpf), we counted melanophores in a dorsal view in a 1 mm^2^ dorsal area (from the edge of the head to edge of the dorsal fin), in the horizontal myoseptum (lateral stripe) and in a ventral view of the entire head. At the early metamorphic (15 dpf) and also the mid metamorphic stages (30 dpf), we counted melanophores in a dorsal view on the head in a 1 mm^2^ dorsal area, in the horizontal myoseptum and in a ventral view of the head and the belly. In adult fish (60 and 210 dpf) melanophores within a 1 mm^2^ area were counted in several positions: in a dorsal view on the head (head area) and on the dorsal area (from the edge of the head to edge of the dorsal fin); in a lateral view, on the stripes 2D, 1D, 1 V and 2 V anterior areas (pectoral to pelvic fin); and finally, in a ventral view of the head and the belly (pectoral to pelvic fin). The dorsal-ventral xanthophore pattern of *asip1* knockout mutant fish was compared with control fish by quantification of pigmented xanthophores in post-metamorphic fish (60 and 210 dpf) (Fig. 4). Selected regions for xanthophore counting were in the dorsal anterior trunk (from the rear edge of the head to front edge of the dorsal fin), and in a ventral view of the belly (from base of pectoral to base of pelvic fin). To analyze the number of melanophores and xanthophores, seven fish per group were anesthetized as before and immersed in 10 mg/ml epinephrine (Sigma) solution for 30 min to contract melanosomes. Fish were photographed on a Leica M165FC stereomicroscope equipped with a Leica DFC310FX camera. Melanophores were counted using ADOBE PHOTOSHOP CS2 software (Adobe Systems Software Adobe Systems Ibérica SL, Barcelona, Spain) and the ImageJ software (National Institutes of Health, NIH, Maryland, USA). Data were statistically evaluated by Student’s *t*-test and data are expressed as mean ± standard error of the mean (SEM). *n* = *7 samples for each count presented*. A *p*-value < 0.05 (asterisks) was considered statistically significant.

### Rescue of CRISPR mediated mutations

Knockout/Transgenic line were obtained by setting up crosses between the CRISPR1-*asip1*.iim08 mutant line and the transgenic reporter line Tg(Xla.Eef1a1:Cau.Asip1)iim05^8^, which ectopically overexpresses *asip1* and produces a dorsal-ventral disruption of pigment pattern phenotype with dorsal skin as pale colored as ventral skin. The offspring were then incrossed to obtain the F2 generation and the *asip1* locus was sequenced to confirm the homozygous knockout mutation (*asip1*^*K.O*.^) that carries the dominant *asip1* transgene. Adult double transgenic/mutant zebrafish (160 dpf) were anesthetized with MS-222 and photographed. Microscope imaging was carried out on a Leica S6D stereomicroscope equipped with a Leica DFC310FX camera.

## Supplementary information

Supplementary information

## References

[1] Millar SE, Miller MW, Stevens ME, Barsh GS (1995). Expression and transgenic studies of the mouse agouti gene provide insight into the mechanisms by which mammalian coat color patterns are generated. Development.

[2] Manceau M, Domingues VS, Mallarino R, Hoekstra HE (2011). The developmental role of agouti in color pattern evolution. Science.

[3] Fujii, R. In *The Physiology of Fishes* (ed. Evans, D.) 535–562 (FL CRC Press, 1993).

[4] Schartl M (2016). What is a vertebrate pigment cell?. Pigment Cell Melanoma Res..

[5] Hirata M, Nakamura K, Kanemaru T, Shibata Y, Kondo S (2003). Pigment cell organization in the hypodermis of zebrafish. Dev. Dyn..

[6] Hirata M, Nakamura K-I, Kondo S (2005). Pigment cell distributions in different tissues of the zebrafish, with special reference to the striped pigment pattern. Dev. Dyn..

[7] Kottler VA, Künstner A, Schartl M (2015). Pheomelanin in fish?. Pigment Cell Melanoma Res..

[8] Ceinos RM, Guillot R, Kelsh RN, Cerdá-Reverter JM, Rotllant J (2015). Pigment patterns in adult fish result from superimposition of two largely independent pigmentation mechanisms. Pigment Cell Melanoma Res..

[9] Frohnhöfer HG, Krauss J, Maischein H-M, Nüsslein-Volhard C (2013). Iridophores and their interactions with other chromatophores are required for stripe formation in zebrafish. Development.

[10] Irion U, Singh AP, Nüsslein-Volhard C (2016). The developmental genetics of vertebrate color pattern formation: lessons from zebrafish. Curr. Top. Dev. Biol..

[11] Cerdá-Reverter JM, Haitina T, Schiöth HB, Peter RE (2005). Gene structure of the goldfish agouti-signaling protein: a putative role in the dorsal-ventral pigment pattern of fish. Endocrinology.

[12] Guillot R, Ceinos RM, Cal R, Rotllant J, Cerdá-Reverter JM (2012). Transient ectopic overexpression of agouti-signalling protein 1 (Asip1) induces pigment anomalies in flatfish. PLoS One.

[13] Cal L (2017). BAC Recombineering of the agouti loci from spotted gar and zebrafish reveals the evolutionary ancestry of dorsal–ventral pigment asymmetry in fish. J. Exp. Zool. Part B Mol. Dev. Evol..

[14] Bassett AR, Tibbit C, Ponting CP, Liu JL (2013). Highly efficient targeted mutagenesis of drosophila with the CRISPR/Cas9 system. Cell Rep..

[15] Manne J, Argeson AC, Siracusa LD (1995). Mechanisms for the pleiotropic effects of the agouti gene. Proc Natl Acad Sci USA.

[16] McNulty JC (2005). Structures of the agouti signaling protein. J. Mol. Biol..

[17] Patel MP (2010). Loop swapped chimeras of the agouti-related protein (agrp) and the agouti signaling protein (ASIP) identify contacts required for melanocortin 1 receptor (MC1R) selectivity and antagonism. J. Mol. Biol..

[18] Kelsh RN (2004). Genetics and evolution of pigment patterns in fish. Pigment Cell Res..

[19] Parichy DM, Elizondo MR, Mills MG, Gordon TN, Engeszer RE (2009). Normal table of postembryonic zebrafish development: staging by externally visible anatomy of the living fish. Dev. Dyn..

[20] Anelli V (2009). Global repression of cancer gene expression in a zebrafish model of melanoma is linked to epigenetic regulation. Zebrafish.

[21] Levesque MP, Krauss J, Koehler C, Boden C, Harris MP (2013). New tools for the identification of developmentally regulated enhancer regions in embryonic and adult zebrafish. Zebrafish.

[22] Michaud EJ, Bultman SJ, Stubbs LJ, Woychik RP (1993). The embryonic lethality of homozygous lethal yellow mice [Ay/Ay] is associated with the disruption of a novel RNA-binding protein. Genes Dev..

[23] Miller MW (1993). Cloning of the mouse agouti gene predicts a secreted protein ubiquitously expressed in mice carrying the lethal yellow mutation. Genes Dev..

[24] Guillot R (2016). Behind melanocortin antagonist overexpression in the zebrafish brain: a behavioral and transcriptomic approach. Horm. Behav..

[25] Sánchez E, Rubio VC, Cerdá-Reverter JM (2010). Molecular and pharmacological characterization of the melanocortin type 1 receptor in the sea bass. Gen. Comp. Endocrinol..

[26] Gross JB, Borowsky R, Tabin CJ (2009). A novel role for Mc1r in the parallel evolution of depigmentation in independent populations of the cavefish Astyanax mexicanus. PLoS Genet..

[27] Parichy DM, Ransom DG, Paw B, Zon LI, Johnson SL (2000). An orthologue of the kit-related gene fms is required for development of neural crest-derived xanthophores and a subpopulation of adult melanocytes in the zebrafish, Danio rerio. Development.

[28] Lopes SS (2008). Leukocyte tyrosine kinase functions in pigment cell development. PLoS Genet..

[29] Fukuzawa T, Ide H (1988). A ventrally localized inhibitor of melanization in Xenopus laevis skin. Dev. Biol..

[30] Bagnara JT, Fukuzawa T (1990). Stimulation of cultured iridophores by amphibian ventral conditioned media. Pigment cell Melanoa Res..

[31] Zuasti A (2002). Melanization stimulating factor (MSF) and melanization inhibiting factor (MIF) in the integument of fish. Microsc. Res. Tech..

[32] Singh AP (2016). Pigment cell progenitors in zebrafish remain multipotent through metamorphosis. Dev. Cell.

[33] Westerfield, M. *The Zebrafish Book. A Guide for the Laboratory Use of Zebrafish (Danio rerio)* (University of Oregon Press, 2007).

[34] Kimmel CB, Ballard WW, Kimmel SR, Ullmann B, Schilling TF (1995). Stages of embryonic development of the zebrafish. Dev. Dyn..

[35] Kettleborough RN (2013). A systematic genome-wide analysis of zebrafish protein-coding gene function. Nature.

[36] Montague, T. G., Cruz, J. M., Gagnon, J. A., Church, G. M. & Valen, E. CHOPCHOP: a CRISPR/Cas9 and TALEN web tool for genome editing. *Nucleic Acids Res*. **42** (2014). 10.1093/nar/gku410 PMC4086086 24861617

