# Supplementary material for: Countershading in zebrafish results from an Asip1 controlled dorsoventral gradient of pigment cell differentiation
Source: Sci Rep. 2019 Mar 5;9:3449. doi: 10.1038/s41598-019-40251-z (PMC6401153; doi:10.1038/s41598-019-40251-z)
Supplement: Supplementary file 1 — Supplementary information [file 41598_2019_40251_MOESM1_ESM.pdf]

Countershading in zebrafish results from an Asip1 controlled dorsoventral gradient of pigment cell differentiation

Laura Cal[a], Paula Suarez-Bregua[a], Pilar Comesaña[a], Jennifer Owen[b], Ingo Braasch[c], Robert Kelsh[b], José Miguel Cerdá-Reverter[d], Josep Rotllant[a]*

SUPPLEMENTARY MATERIAL

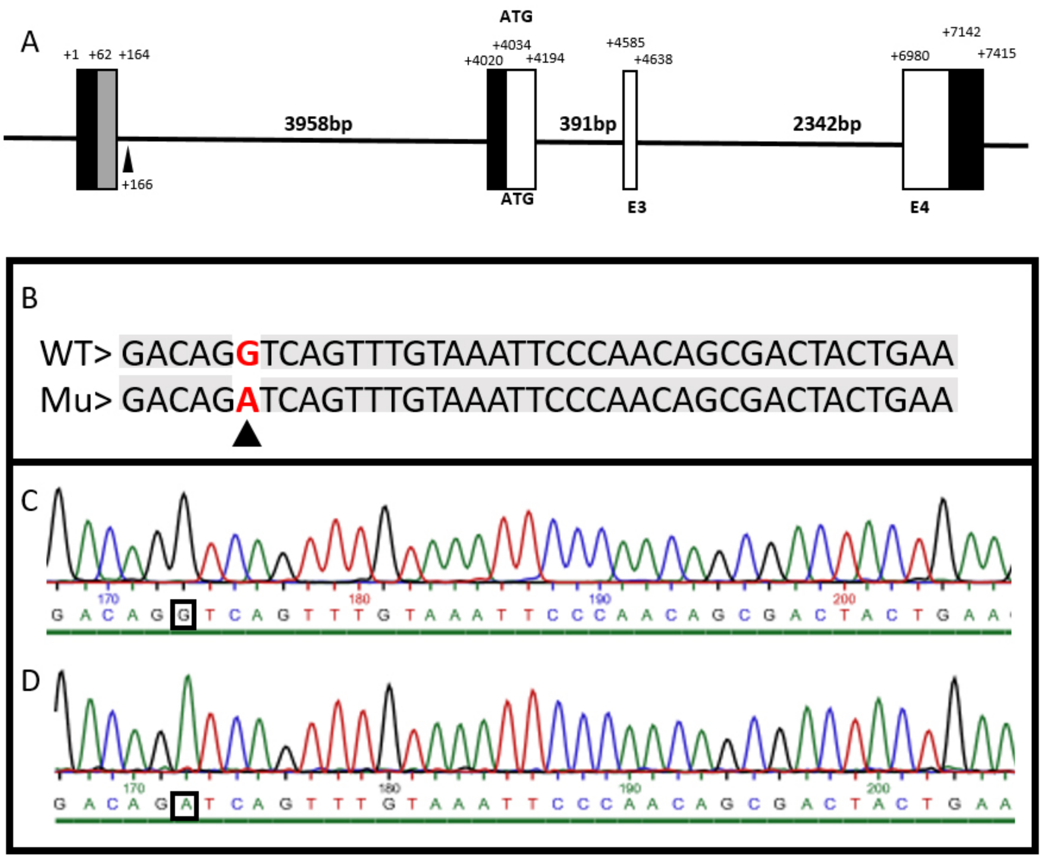

Supplementary Figure 1. *asip1(sa13992)* mutation (A) Scheme of the *asip1* gene showing the position of the point mutation (black arrowhead) as in Figure 1A. Wellcome Sanger institute suggests this point mutation to be located at an 'essential splice site.' Coding exons are represented as white boxes and 5' UTR and 3'UTR are shown as black boxes. An alternative 3' extension to the first exon as suggested by UGENE is indicated in grey. The point mutation is located 2 bp downstream of this alternative exon. (B) Sequence of point mutation in the *asip1* locus. The first line shows the wild-type sequence

with the position of the point mutation marked with a black arrowhead. Next line shows the sequence of mutant *asip1(sa13992)*. (C) Genomic sequence obtained for wild-type fish. (D) Genomic sequence obtained for homozygous *asip1(sa13992)* fish.

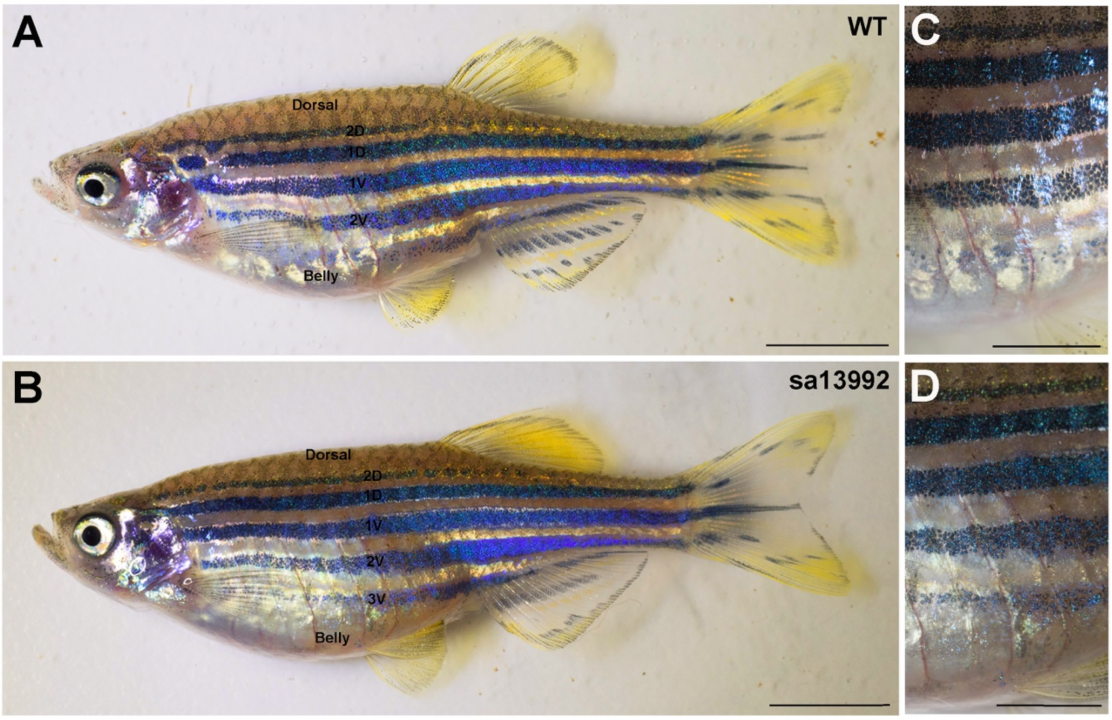

Supplementary Figure 2. Adult dorso-ventral countershading in *asip1(sa13992)* mutant. Lateral (A, B), anterior-lateral (C, D), views of 180 dpf *asip1(sa13992)* mutant (A, C) and WT(B, D) zebrafish. (A, B) The pigment pattern of WT zebrafish is a striped pigment pattern with dark stripes and light interstripes. Each dark stripe is named with a code: two primary stripes are called 1D and 1V, and the two secondary stripes are named 2D and 2V. The *asip1(sa13992)* mutants display a more pronounced 3V dark stripe. Scale bar: (A,B) 5 mm, (CD) 10 mm.
